# A preliminary study on the relationship between central auditory processing and childhood primary headaches in the intercritical phase

**DOI:** 10.1186/1129-2377-14-69

**Published:** 2013-08-09

**Authors:** Antonella Ciriaco, Angelo Russo, Daniele Monzani, Elisabetta Genovese, Paola Benincasa, Ernesto Caffo, Luigi Alberto Pini

**Affiliations:** 1 Headache and Drug Abuse Inter-Dep Research Centre, University of Modena and Reggio Emilia, via del Pozzo 71, Modena, Italy; 2 Child Neurology and Psychiatry Unit, University of Modena and Reggio Emilia, via del Pozzo 71, 41124 Modena, Italy; 3 Audiology Unit, Department of Diagnostic Medicine, Clinical and Public Health, University of Modena and Reggio Emilia, via del Pozzo 71, Modena, Italy; 4 Otolaryngology Department, Hospital of Carpi, Via Guido Molinari 2, Carpi, Italy

**Keywords:** Childhood, Primary headache, Speech perception, Audiometry, Auditory processing

## Abstract

**Background:**

Recently, an increasing number of articles have appeared on central auditory processing disorders, but in the literature there is only one study that evaluated the possible correlation between migraine in the critical phase and central auditory processing. The aim of our study was to assess the correlation between auditory processing information and childhood primary headaches in the intercritical phase.

**Methods:**

This is an observational study. We enrolled 54 patients, 30 with primary headache (migraine and tension headache) and 24 normal controls, matched for sex and age. The mean age at first observation was 9 years 10 months; the duration of observational follow-up was 2 years. Both groups had normal audiological and neurological profiles, normal peripheral hearing acuity and normal cognitive and behavioral skills. We excluded patients who had undergone pharmacological prophylactic treatment for headaches in the 6 months preceding the study and subjects with a frequency of headache lower than one every two months. After enrolment, both groups were analyzed with a computerized test battery for Speech Perception Tests in silence and in noise background to assess speech perception disabilities. In addition, with a test battery of Speech Perception Tests, we compared patients with migraines and tension-type headaches. The non-parametric χ2 test, the Mann–Whitney U-test and the Wilcoxon signed ranks test were used for statistical analysis. P-values <0.05 were considered significant and STATA 10 software was used for statistical analyses.

**Results:**

Our results showed that patients with primary headache (migraine and tension-type headache), had a deficit of auditory processing in noisy background compared to control cases, but we found no significant differences when we compared patients with migraine and tension-type headache.

**Conclusions:**

This is a work in progress and further studies are needed to assess the relationship between the impairment of auditory processing and primary headache, not only to improve the diagnostic approach to primary headache, but also to improve therapeutic intervention.

## Background

Headache, a frequently occurring disorder in children and adolescents, is the third most common illness-related cause of absence from school, resulting in substantial impairment among pediatric patients [1-4]. The majority of headaches in children and adolescents are not associated with structural or organic disease [5-7]. Migraines and tension-type headaches are chronic disorders often starting in childhood, and about 50% of children and adolescents with migraine continue to suffer from the disorder into adulthood [1,8].

A tendency to fidget and to be easily distracted, poor concentration and poor academic achievement are characteristics of children both with the primary headache and with the auditory processing disorders, like difficulty in understanding conversation amid background noise [5-7]. Auditory processing refers to the electrical transformation and transmission of the auditory signal after it leaves the mechanical processes of the outer, middle, and inner ear [9,10]. The auditory signal is transmitted by the auditory nerve, a branch of the eighth cranial nerve, via the ascending auditory pathways, to term in the auditory cortex and cerebellum [9-11]. In recent years, an increasing number of articles have appeared on central auditory processing disorders [12-21]. The impairment of auditory processing should not cause a disorder of attention, cognition, or language, although these may be comorbid [9,12-21]. The behavioral overlap of co-morbid diagnoses is due to the widely distributed networks with which central auditory pathways are linked, in bottom-up, top-down, and reciprocal loop processes [5]. The diagnosis of an auditory processing disorder is made after it has been established by audiological evaluation that a patient has normal peripheral hearing, and this can be detected by valid and reliable electrophysiological, imaging, and behavioural assessment techniques [11,21]. Behavioral tests of auditory processing demonstrate good correlations with electrophysiological measures for distinguishing auditory processing disorder as a separate diagnostic entity if cognitive and/or behavioural deficits are excluded in the evaluation of patients [13-21]. Several authors have shown that migraineurs, during the intercritical phase, had a lack of habituation, or even potentiation, of cortical evoked potentials during repetitive stimulation and have a strong intensity dependence of auditory evoked potentials [22-24]. One recent study evaluated the possible relationship between migraine, during attacks, and auditory processing; the authors concluded that the function of the neural substrates, responsible for auditory processing information, is impaired during the migraine attack [25]. The aim of this study was to evaluate the correlation between auditory processing information and childhood primary headaches in the intercritical phase.

## Method

In this observational study we enrolled 54 patients, between the ages of 6 and 12 years, referred to the Polyclinic hospital of Modena from 2009 to 2011. Among these 54 subjects , we studied 30 cases (14 male and 16 female) who were patients of the Headache and Drug Abuse Inter-Department Research Centre of University of Modena and Reggio Emilia and selected, from a database of healthy children of the Pediatric Clinic of Polyclinic of Modena, 24 normal controls, that were matched for sex and age. We divided the case series with primary headache into two groups: the first included patients with migraine, the second included patients with tension-type headache (Table 1); for both groups the diagnosis of primary headache was made according to the International Classification of Headache Disorders 2nd edition criteria (ICDH-II 2004). In addition, we also considered some additional diagnostic criteria, such as familiarity, behavior during the attacks, presence of osmophobia and trigger factors, to facilitate the differential diagnosis between migraine and tension type headache in children. Moreover, we applied the ICDH-II criteria for headache diagnosis, taking into account also suggestions inserted in Appendix section of the International Classification of Functioning, Disability and Health II [26]. The mean age at first observation was 9 years 10 months; the duration of observational follow-up was 2 years. To be enrolled, both the group with primary headache and the control group had to have a normal audiological and neurological profile, normal peripheral hearing acuity and normal cognitive and behavioral skills. Furthermore, we excluded patients who had undergone pharmacological prophylactic treatment for headache in the 6 months preceding the study, subjects with a frequency of headache lower than one every two months and patients with primary headache attack in progress. After enrolment, both patients with primary headache and healthy children were analyzed with a computerized test battery of Speech Perception Tests in silence and in noise background to assess speech perception disabilities. In this last test, three word lists of 25 words were used: the first was composed of nonsense words, the second of bisyllabic phonemically balanced words, and the third was a list of everyday sentences composed of bisyllabic phonemically balanced words. The results were evaluated as a percentage of correct answers for each word of the test lists. The use of a recorded voice in the automatic administration of Speech Perception Tests reduces speaker-related variables (e.g. voice characteristics) and listener-related variables (such as the degree of attention). The non-parametric χ2 test was used to compare the observed frequencies of patients’ pathological scores with those predicted for the normal population. Since the scores obtained for most dependent variables are not distributed normally, the Mann–Whitney U-test was used to compare migraine and tension-type headache in behavioral measures, and the Wilcoxon signed ranks test was used to compare repeated measures within the same sample. All the statistical analyses were two-tailed and p-values of ≤0.05 were considered significant. STATA 10 software was used for statistical analyses. I will inform you that the local Ethic Committee approved the study in date June 16th 2009, registered with protocol number 1921. The study code was NOC09, and this study does not have any Eudrug number being an observational study.

**Table 1:** **Age and gender characteristics** (**clinical characteristics**) **of children and adolescent with headache and normal control**

	**Headache group**						**Control group *N = 24***	
	**TTH**		**M**		**All**			
	***N = 11***		***N = 19***		***N = 30***			
**Age** (*mean* ± *SD*)	9.95 ± 1.97		10.10 ± 1.57		10.02 ± 1.74		9.7 ± 1.7	
**Male**	6	(55%)	8	(42%)	14	(47%)	10	(42%)
**Female**	5	(45%)	11	(58%)	16	(53%)	14	(58%)

*M* migraine, *TTH* tension-type headache, *N* number of patients. Data are presented as percentage ± SD of positive exact performance.

## Results and discussion

Initially, we enrolled 60 patients, 30 with primary headache and 30 control cases, whereas the case set of the control group decreased in number during the follow-up because six patients dropped out of the study for personal and family reasons. Thus, the results refer to a population of 54 patients (30 with primary headache and 24 control cases). All subjects had normal audiological and neurological profiles, normal peripheral hearing acuity and normal cognitive and behavioral skills. Audiometry with air- and bone-conduction thresholds was within the norm (Headache group: 10.9 Hz ± 1.8 DS; Control group: 10.5 Hz ± 2.5 DS) and there were no statistical differences hearing threshold levels among patients with primary headache and the control group (p = 0.704). In Speech Perception Tests, in silent backgrounds, all patients got 100% of the responses correct (Tables 1, 2, and 3). During Speech Perception Tests in a noise background, however, the primary headache group (migraine and tension headache) presented more incorrect response than the control cases (Table 2). In fact, we found that the group with primary headache showed a significant deficit compared to the control cases in all items performed, in auditory processing in noise background, in the repetition of non-sense bi-syllabic words (p < 0.001), bisyllabic phonemically balanced words (p = 0.026), and a list of everyday sentences composed of bisyllabic phonemically balanced words (p < 0.001). In addition, we found that patients with migraine and those with tension-type headache (separately) had a significant deficit in auditory processing compared to control cases in the item of Speech Perception Tests, that evaluated the repetition of non-sense bi-syllabic words (p < 0.001) and the repetition of a list of everyday sentences composed of bisyllabic phonemically balanced words (p < 0.001), but not in those that analyze the repetition of bisyllabic phonemically balanced words (p = 0.85) (Table 3). Finally, we found no significant differences in any of the items of the Speech Perception Tests, in a noise background, in the comparison between patients with migraine and tension-type headache (p > 0.05) (Table 4). Our results showed that patients with primary headache (migraine and tension headache), had a deficit of auditory processing in noisy background compared to control cases, but we found no significant differences when we compared patients with migraine and tension-type headache. Several authors claim that the comorbid diagnoses of the impairment of auditory processing, and the disorder of attention, cognition, or language, are due to the widely distributed networks with which central auditory pathways are linked, in bottom-up, top-down, and reciprocal loop processes [9]. Thus, it is reasonable to believe that the same hypothesis could be considered with regard to relations between primary headache and auditory processing. In this regard, interesting results have been obtained by different authors who have shown that migraineurs, during the intercritical phase, had a lack of habituation, or even potentiation, of cortical evoked potentials during repetitive stimulation and a strong intensity dependence of auditory evoked potentials, and that both these abnormalities in information processing could be due to a hypofunction of brainstem serotonergic projections to sensory cortices, emphasizing the link or overlap with the pathogenesis of primary headache. Furthermore, a recent study, showing a deficit in auditory processing during the migraine attack, reinforces this hypothesis, and the authors indicate that the function of neural substrates, responsible for different stages of auditory information processing, is impaired also during the acute migraine [25]. Further studies are needed.

**Table 2:** Correlation between primary headaches and control group in auditory processing

		**All headache**	**Control group**	**P value**
		***N*** **=** ***30***	***N*** **=** ***24***	
**Speech perception test in silence**	**VCV**	100 ± 0	100 ± 0	
	**Bisill**	100 ± 0	100 ± 0	
	**Fr**	100 ± 0	100 ± 0	
**Speech perception test in noise**	**VCV**	**64.6 ± 10.7**	**81.2 ± 9**	**=0.0001**
	**Bisill**	**61.8 ± 19.8**	**74.8 ± 19.3**	**=0.0026**
	**Fr**	**86.7 ± 11.9**	**97.2 ± 4.9**	**=0.0001**

*VCV* list composed of nonsense words, *BiSi* list with bisyllabic phonemically balanced words, *Fr* list of everyday sentences composed of bisyllabic phonemically balanced words. Data are presented as percentage ± SD of positive exact performance.

**Table 3:** **Correlation between migraine**, **tension**-**type headache and control group in auditory processing**

		**TTH**	**M**	**Control group**	**P value**
		***N*** **=** ***11***	***N*** **=** ***19***	***N*** **=** ***24***	
**Speech perception test in silence**	**VCV**	100 ± 0	100 ± 0	100 ± 0	
	**Bisill**	100 ± 0	100 ± 0	100 ± 0	
	**Fr**	100 ± 0	100 ± 0	100 ± 0	
**Speech perception test in noise**	**VCV**	**65.78 ± 10.7**	**62.6 ± 11.05**	**81.2 ± 9**	**=0.0001**
	**Bisill**	**63.89 ± 16.49**	**58 ± 25.29**	**74.8 ± 19.3**	**n.s.**
	**Fr**	**88.67 ± 7.48**	**83.2 ± 17.36**	**97.2 ± 4.9**	**=0.0001**

*VCV* list composed of nonsense words, *BiSi* list with bisyllabic phonemically balanced words, *Fr* list of everyday sentences composed of bisyllabic phonemically balanced words, *M* migraine, *TTH* tension-type headache. Data are presented as percentage ± SD of positive exact performance.

**Table 4:** **Correlation between migraine and tension**-**type headache in auditory processing**

		**TTH**	**M**	**P value**
		***N*** **=** ***11***	***N*** **=** ***19***	
**Speech perception test in silence**	**VCV**	100 ± 0	100 ± 0	
	**Bisill**	100 ± 0	100 ± 0	
	**Fr**	100 ± 0	100 ± 0	
**Speech perception test in noise**	**VCV**	**65.78 ± 10.7**	**62.6 ± 11.05**	**n.s.**
	**Bisill**	**63.89 ± 16.49**	**58 ± 25.29**	**n.s.**
	**Fr**	**88.67 ± 7.48**	**83.2 ± 17.36**	**n.s.**

*VCV* list composed of nonsense words, *BiSi* list with bisyllabic phonemically balanced words, *Fr* list of everyday sentences composed of bisyllabic phonemically balanced words, *M* migraine, *TTH* tension-type headache. Data are presented as percentage ± SD of positive exact performance.

## Conclusions

In conclusion, our study suggests that the impairment of auditory processing, through a mechanism not yet well-known, is likely to be correlated to the primary headache, regardless of whether it was a migraine or a tension-type headache. This is a work in progress, in fact we intend to continue the study by expanding the case series, comparing the results of the Speech Perception Tests with the neurophysiological investigations, and using the study of functional neuroimaging, with the aim of investigating more thoroughly the possible overlaps between the primary headache and central auditory processing. Further studies are needed, therefore, not only to improve the diagnostic approach to patients with primary headache, but also to improve both pharmacological and non-pharmacological therapeutic intervention.

### Consent

The authors received no financial support for the research, authorship, and/or publication of this article. Consent was obtained from the children’s parents for the preparation and publication of this case report.

## Competing interests

The authors declare that there are no potential conflicts of interest with respect to the research, authorship, and/or publication of this article.

## Authors’ contributions

AC had full access to all of the data in the study, takes responsibility for the integrity of data and the accuracy of the data analysis. AR had full access to all of the data in the study, discussed the data interpretation and wrote the manuscript. PM carried out the audiological evaluation. EG supervised the audiological evaluation and discussed the data interpretation. PB carried out the speech evaluation. EC revised the first draft of the manuscript. LAP was a key member of the clinical team, co-designed the study and discussed interpretation of the data. All authors have contributed to, seen, and approved the manuscript.
